# Molecular Analysis of *Acinetobacter baumannii* Strains Isolated in Lebanon Using Four Different Typing Methods

**DOI:** 10.1371/journal.pone.0115969

**Published:** 2014-12-26

**Authors:** Rayane Rafei, Fouad Dabboussi, Monzer Hamze, Matthieu Eveillard, Carole Lemarié, Marie-Pierre Gaultier, Hassan Mallat, Rima Moghnieh, Rola Husni-Samaha, Marie-Laure Joly-Guillou, Marie Kempf

**Affiliations:** 1 L'UNAM Université, Université d'Angers, Groupe d'Etude des Interactions Hôte-Pathogène, UPRES EA3142, Institut de Biologie en Santé – IRIS, CHU, Angers cedex, France; 2 Laboratoire de Santé et environnement, Centre AZM pour la recherche en Biotechnologie et ses applications, Université Libanaise, Tripoli, Liban; 3 Laboratoire de Bactériologie, Institut de Biologie en Santé - PBH, CHU, Angers cedex, France; 4 Ain wa zein Hospital, Shouf, Lebanon; 5 Division of Infectious Diseases, Department of Internal Medicine, Lebanese American University Medical Center Rizk Hospital, Beirut, Lebanon; George Mason University, United States of America

## Abstract

This study analyzed 42 *Acinetobacter baumannii* strains collected between 2009–2012 from different hospitals in Beyrouth and North Lebanon to better understand the epidemiology and carbapenem resistance mechanisms in our collection and to compare the robustness of pulsed field gel electrophoresis (PFGE), multilocus sequence typing (MLST), repetitive sequence-based PCR (rep-PCR) and *bla*_OXA-51_ sequence-based typing (SBT). Among 31 carbapenem resistant strains, we have detected three carbapenem resistance genes: 28 carried the *bla*_OXA-23_ gene, 1 the *bla*_OXA-24_ gene and 2 strains the *bla*_OXA-58_ gene. This is the first detection of *bla*_OXA-23_ and *bla*_OXA-24_ in Lebanon. PFGE identified 11 types and was the most discriminating technique followed by rep-PCR (9 types), *bla*_OXA-51_ SBT (8 types) and MLST (7 types). The PFGE type A'/ST2 was the dominant genotype in our collection present in Beyrouth and North Lebanon. The clustering agreement between all techniques was measured by adjust Wallace coefficient. An overall agreement has been demonstrated. High values of adjust Wallace coefficient were found with followed combinations: PFGE to predict MLST types  = 100%, PFGE to predict *bla*_OXA-51_ SBT = 100%, *bla*_OXA-51_ SBT to predict MLST = 100%, MLST to predict *bla*_OXA-51_ SBT = 84.7%, rep-PCR to predict MLST = 81.5%, PFGE to predict rep-PCR = 69% and rep-PCR to predict *bla*_OXA-51_ SBT = 67.2%. PFGE and MLST are gold standard methods for outbreaks investigation and population structure studies respectively. Otherwise, these two techniques are technically, time and cost demanding. We recommend the use of *bla*_OXA-51_ SBT as first typing method to screen isolates and assign them to their corresponding clonal lineages. Repetitive sequence-based PCR is a rapid tool to access outbreaks but careful interpretation of results must be always performed.

## Introduction

*Acinetobacter baumannii* is an opportunistic gram negative pathogen involved in a wide number of nosocomial infections like ventilator-associated pneumonia, bloodstream, urinary tract, wound and meningitis infections frequently associated with a high rate of mortality and morbidity [1]. Outbreaks have been intensively documented worldwide and are usually caused by multidrug resistant strains and more and more carbapenem resistant strains [2], [3]. These outbreaks strains mainly belonged to three international clones I, II and III (previously called as European clones [4], [5]), but also to different clonal lineages. Karah *et al.* [2] analyzed the MLST-based global population structure of *A. baumannii* on 496 isolates. They showed the presence of 26 clones and among them, 18 were international clones and 8 European or Asian restricted clones. The International clone II was the major clone reported in 34 countries in Europe, Asia, Africa Australia, USA, and South America.

To track and monitor these outbreaks, denote strain relatedness and assign an outbreak strain to its corresponding clonal lineage, many typing methods with different intrinsic degrees of resolution are proposed [3] such as pulsed field gel electrophoresis (PFGE) [6], repetitive-sequence-based PCR (rep-PCR) [7], amplified fragment length polymorphism (AFLP) [8], multilocus sequence typing (MLST) [9], [10], 3-locus sequence typing (3-LST) [11], *bla*_OXA-51_ sequence-based typing (SBT) [12] or Multiple-Locus Variable number of tandem repeat Analysis (MLVA) [13]. Selection of an appropriate genotyping technique is not always easy and depends to the studied objectives. Many authors emphasized the great need to validate the application of each method as well as to harmonize different typing methods by reference networks [3], [14]. Among these methods, PFGE is still considered the current gold standard for *A. baumannii* outbreak investigation at local scale [3]. MLST has a discriminatory power lesser than PFGE and is regarded as the gold standard for large epidemiological and population structure studies. For *A. baumannii*, two MLST schemes have been proposed: Bartual's MLST [9] and Pasteur's MLST [10]. DiversiLab is a semi-automated form of rep-PCR with a comparable discriminatory power to PFGE [15]. The identification of eight international clones is one of the remarkable advantages of this system [7]. *bla*_OXA-51_ SBT has been proposed as a single-locus based typing [12] with a similar discriminatory power to rep-PCR [16] and Bartual and Pasteur's MLST [12], [17].

In Lebanon, there have been limited reports studying only local outbreaks in Beyrouth between 2004 and 2007 where *bla*_OXA-58_ was the only carbapenem resistance gene identified [18]–[20]. Recently, we have detected four *bla*_NDM-1_ producing *A. baumannii* isolated in Tripoli, Northern Lebanon from Syrian civilians wounded during Syrian war [21].

The present study has a double aim: firstly to compare the performance and effectiveness of four epidemiological typing methods (PFGE, rep-PCR, MLST and *bla*_OXA-51-like_ SBT), and secondary to get primary results on circulating clones and carbapenem resistance mechanisms in Lebanon by analysis of 42 non duplicate strains conserved on Azm center for research on biotechnology and its application (Lebanese university) and collected between 2006–2012 from different hospitals in Beyrouth and North Lebanon.

## Results

### Identification

Forty-two strains were confirmed as *A. baumannii* by molecular techniques. These strains were isolated in different hospitals in Beyrouth (24 strains) and North Lebanon (18 strains) from various clinical specimens between 2009 and 2012 except one strain isolated in 2006 (Table 1). Beyrouth strains were isolated during epidemiological contexts.

**Table 1:** Origin and repartition of strains used in this study.

Strain ID	Hospital	City	Department	Gender	Age	Period study	Sample origin
8	Nini	Tripoli	ICU	M	70	2009	tracheal aspirate
9	RHH	Beyrouth	ICU	M	79	2011	blood
13	RHH	Beyrouth	NA	NA	NA	2011	NA
15	RHH	Beyrouth	ICU	M	68	2011	blood
17	Nini	Tripoli	Cardiology	M	48	2009	urine
19	RHH	Beyrouth	NA	NA	NA	2011	NA
20	RHH	Beyrouth	ICU	F	69	2011	blood
21	RHH	Beyrouth	NA	NA	NA	2011	NA
22	RHH	Beyrouth	NA	NA	NA	2011	NA
23	AWH	Beyrouth	NA	M	89	2011	rectum
24	RHH	Beyrouth	ICU	M	54	2011	blood
25	RHH	Beyrouth	NA	NA	NA	2011	NA
28	Nini	Tripoli	Outpatient	F	51	2011	urine
29	Nini	Tripoli	NA	F	22	2011	urine
30	RHH	Beyrouth	NA	NA	NA	2011	NA
31	Nini	Tripoli	ICU	M	72	2011	tracheal aspirate
34	RHH	Beyrouth	NA	NA	NA	2011	NA
35	Nini	Tripoli	Outpatient	M	37	2011	bedsore
36	Nini	Tripoli	ICU	M	74	2012	tracheal aspirate
37	AWH	Beyrouth	NA	M	89	2011	throat
38	Nini	Tripoli	Cardiology	M	57	2006	tracheal aspirate
40	Nini	Tripoli	ICU	M	20	2012	blood
41	RHH	Beyrouth	NA	NA	NA	2011	NA
45	RHH	Beyrouth	NA	NA	NA	2011	NA
46	Monla	Tripoli	NA	M	37	2012	chest drain
47	AWH	Beyrouth	NA	F	65	2011	sputum
48	RHH	Beyrouth	NA	M	74	2011	blood
49	RHH	Beyrouth	NA	NA	NA	2011	NA
50	RHH	Beyrouth	NA	NA	NA	2011	NA
51	RHH	Beyrouth	NA	NA	NA	2011	NA
52	RHH	Beyrouth	NA	NA	NA	2011	NA
53	Nini	Tripoli	Maternity	F	25	2010	urine
56	Nini	Tripoli	ICU	F	79	2011	bronchial aspirate
58	AWH	Beyrouth	NA	F	39	2011	sputum
59	RHH	Beyrouth	NA	F	70	2011	blood
60	RHH	Beyrouth	NA	NA	NA	2011	NA
62	Nini	Tripoli	Outpatient	F	74	2012	urine
63	Rahal	Akkar	Internal medicine	M	46	2012	wound
65	TGH	Tripoli	Internal medicine	M	27	2012	wound
66	TGH	Tripoli	ICU	M	29	2012	sputum
67	TGH	Tripoli	Internal medicine	F	38	2012	abdomen
68	TGH	Tripoli	Internal medicine	M	29	2012	sputum

AWH: Ain Wazein Hospital; RHH: Rafic Hariri Hospital; TGH: Tripoli Governmental Hospital; ICU: Intensive care unit; NA: not available.

### Carbapenem resistance mechanisms

**Figure 1:**
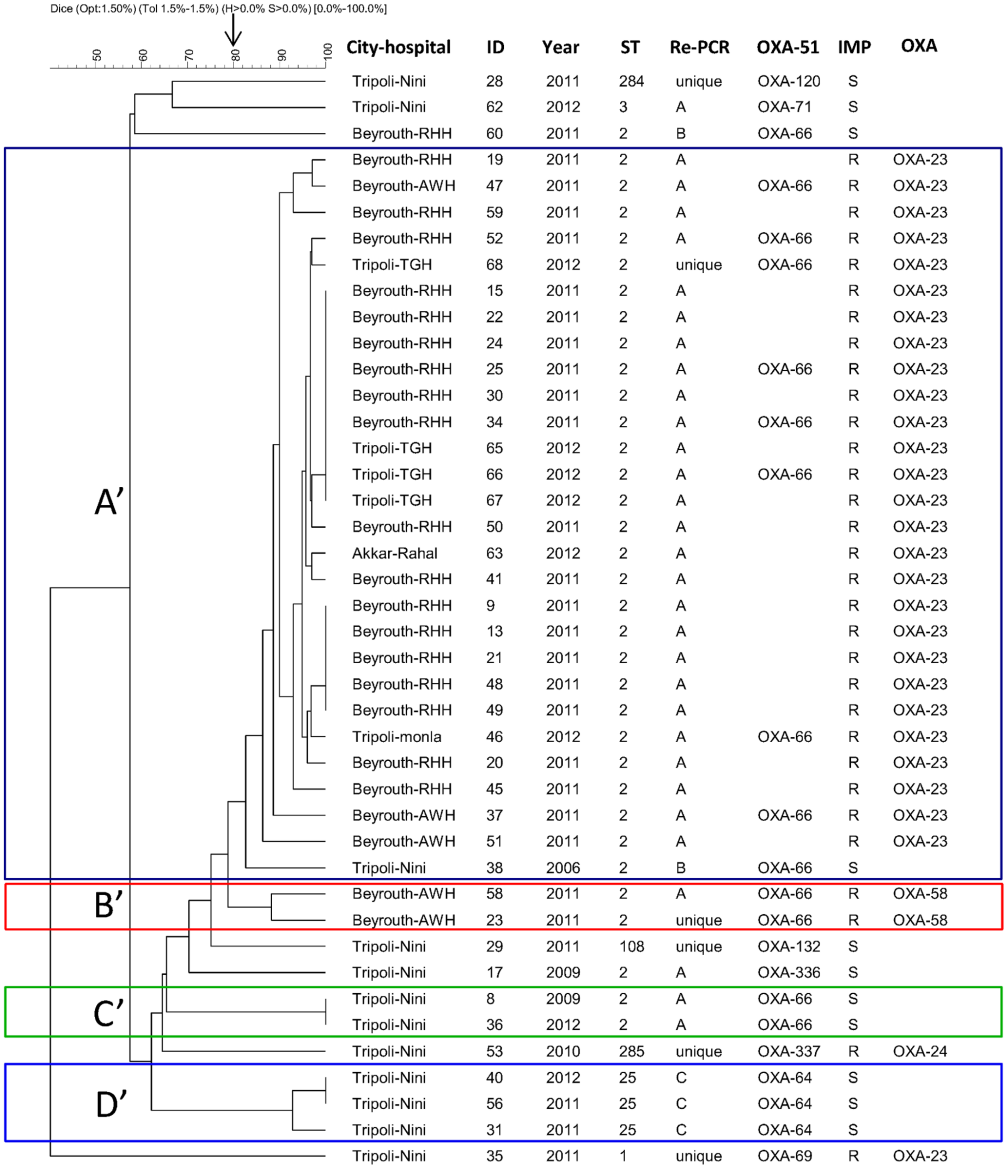
PFGE, MLST, DiversiLab and *bla*_OXA-51-like_ comparison results for 42 *A. baumannii* strains. Dendrogram was generated by cluster analysis of PFGE fingerprinting patterns. Arrow shows the adopted cut-off (80%) for definition of PFGE type. ID: Sample number, rep-PCR for rep-PCR type, IMP: imipenem susceptibility (S for susceptible and R for resistant), OXA: oxacillinase responsible for carbapenem resistance phenotype.

Thirty-one strains showed carbapenem resistance phenotypes (Fig. 1). Among these strains, 28 harbored a *bla*_OXA-23_ gene, 2 a *bla*_OXA-58_ gene, and one a *bla*_OXA-24_ gene. No acquired *bla*_ndm-1_ or *bla*_OXA-143_ has been detected. IS*Aba1* was present in 37 strains. All carbapenem resistant strains except one (strain 53, *bla*_OXA-24_ positive) had this sequence in their genomes whereas 7 carbapenem susceptible strains only had this sequence. The research of IS*Aba1* presence before both *bla*_OXA-51_ and *bla*_OXA-23_ genes revealed its insertion upstream *bla*_OXA-23_ in *bla*_OXA-23_ producing strains but not upstream *bla*_OXA-51_ gene in both carbapenem resistant or susceptible strains. This insertion explains the high level of resistance to carbapenems (imipenem MIC>32, meropenem MIC>32, doripenem 12<MIC>32) for all *bla*_OXA-23_ producing *A. baumannii* strains. The *bla*_OXA-24_-producing strain (strain 53) had MIC: 8 mg/l, 16 mg/l, 16 mg/l for imipenem, meropenem and doripenem respectively, whereas the two *bla*_OXA-58_ - producing strains (strain 58 and 23) showed low level of carbapenem resistance: (4; 8 mg/l), (2; 4 mg/l), (2; 3 mg/l) for imipenem, meropenem and doripenem respectively.

### Epidemiological typing

#### PFGE

Using ≥80% similarity cut-off as a threshold, PFGE classified our strains in 11 types: 4 clusters (A' to D') and 7 unique profiles (Fig. 1). Cluster A' comprised 28 strains, 27 were *bla*_OXA-23_ producing strains and one an imipenem susceptible strain. This cluster contained strains from Beyrouth and North Lebanon. Cluster B' contained two *bla*_OXA-58_ producing strains that belonged to the same hospital. Cluster C' and D' contained two and three imipenem susceptible strains respectively.

#### MLST

MLST typing was performed on all strains. Seven ST(s) were identified (Fig. 1), two of which were novel and assigned as 284 and 285 by MLST Pasteur. The ST2 was the most predominant, present in 34 strains, followed by ST25, present in 3 strains; other ST(s) were present sporadically in our collection. The eBUSRT analysis of our ST(s) with all identified ST(s) in MLST database (28.03.2014) showed that ST 1, 2, 3, 25, 284 belonged to CC 1, 2, 3, 25 and 33 respectively, whereas ST285 was a singleton, and ST108 shared similarity with ST112 (we did not assign a name for this complex because no more of 2 ST(s) have been yet identified). Interestingly, our novel ST284 was a SLV of ST33 (the founder of CC33).

#### rep-PCR

Using ≥95% similarity cut-off as a threshold, rep-PCR identified 9 types (Fig. 1): 3 clusters (cluster A to C) and 6 unique profiles (ungrouped strains). Cluster A was the major cluster which contained 31 strains, whereas cluster B and C contained 2 and 3 strains respectively. Cluster A grouped only strains belonging to clone EU II except one strain (strain 62) belonging to EU clone III. This cluster comprised strains from different hospitals in Beyrouth (22 strains) and from North Lebanon (9 strains). Cluster B comprised two imipenem susceptible strains from Tripoli and Beyrouth, while cluster C contained all strains belonging to ST25 and coming from one hospital in Tripoli. Two unclustered strains belonged to ST2 while the remaining strains belonged to different ST(s).

#### *bla*_OXA-51-like_ sequence-based typing

*bla*_OXA-51_ SBT has been performed with randomly selected strains from rep-PCR type A, and for all remaining strains. Notably, *bla*_OXA-51_ SBT correctly identified our strains. All strains belonging to ST2 (CC2) carried the *bla*_OXA-66_ gene, except one strain (strain 17) which was a colistin resistant one and which carried a single amino acid variant of OXA-66 described for the first time in this study (OXA-336, KF048907). The *bla*_OXA-336_ had a non-synonymous mutation from *bla*_OXA-66_ at nucleotide 518 (thymine became adenine) which led to the substitution of isoleucine by asparagine at amino acid 173. For the other strains, each ST had a unique OXA-51 type: ST1 carried OXA-69, ST3 OXA -71, ST25 OXA-64, ST108 OXA-132, ST284 OXA-120, and ST285 a new OXA (OXA-337, KF048908).

#### Comparison of the four typing methods

The visual analysis of the collection (Fig. 1) showed an overall agreement between the different techniques, although some discrepancies have been noticed. The adjusted Wallace coefficient analysis (Table 2) revealed that all ST(s) obtained by MLST were predicted at 100% level by PFGE and *bla*_OXA-51_ SBT and at 81.5% by rep-PCR. Conversely, MLST was unable to predict PFGE and rep-PCR types, but able to well predict *bla*_OXA-51_ sequences (84.7%). As expected, PFGE types were not predicted by any other technique. In contrast, PFGE predicted at 100% level all ST(s) and *bla*_OXA-51_ sequences and at 69% rep-PCR types. Rep-PCR types were well predicted (69.0%) by PFGE, but not predicted neither by MLST nor by *bla*_OXA-51_ SBT. In contrast, rep-PCR predicted the ST(s) at 81.5% and *bla*_OXA-51_ at 67.2% but was unable to predict PFGE types. Finally for *bla*_OXA-51_ SBT, we have assumed that isolates belonging to ST2 and for which we didn't perform *bla*_OXA-51_ sequencing carried *bla*_OXA-66_ [17]. *bla*_OXA-51_ sequences were all predicted by PFGE, and at 84. 7% and 67.2% by MLST and rep-PCR respectively. Conversely *bla*_OXA-51_ SBT was able to predict all ST(s), but not PFGE and rep-PCR types.

**Table 2:** Concordance between the studied typing techniques using adjusted Wallace coefficient (95% confidence interval).

	PFGE (cut-off 80%)	rep-PCR (cut-off 95%)	MLST	bla_OXA-51_ SBT
**PFGE**		0.690 (0.313–1.000)	1.000 (1.000–1.000)	1.000 (1.000–1.000)
**rep-PCR**	0.462 (0.086–0.838)		0.815 (0.473–1.000)	0.672 (0.259–1.000)
**MLST**	0.422 (0.058–0.786)	0.513 (0.107–0.919)		0.847 (0.564–1.000)
**bla_OXA-51_ SBT**	0.498 (0.142–0.854)	0.499 (0.085–0.914)	1.000 (1.000–1.000)	

## Discussion

This is the first study in Lebanon providing data about clonality and carbapenem resistance mechanisms of a set of isolates recovered from different hospitals in Beyrouth and North Lebanon. However, this study does not illustrate the overall *A. baumannii* molecular epidemiology in this country because it does not contain prospectively collected isolates from different hospitals in different Lebanese provinces.

Currently, worldwide carbapenem resistant strains are mostly associated with international clone 2 [2], with *bla*_OXA-23_ as the main carbapenem resistance mechanism [22]–[24]. Our results stick well to the global situation where the majority of carbapenem resistant strains belonged to ST2, but only two imipenem resistant strains in our collection belonged to ST1 and ST285 (Fig. 1). In several countries, *bla*_OXA-58_ [25] or *bla*_OXA-24_ [26] have been progressively replaced by *bla*_OXA-23_. In Beyrouth-Lebanon, the studied outbreaks between 2004–2007 [18]–[20] were caused by the three international clones (1 to 3) producing OXA-58 as a main carbapenem resistance mechanism. In the present study, we detected *bla*_OXA-58_ in two strains from a hospital in Beyrouth, but *bla*_OXA-23_ seemed to be an emerging carbapenemase in Beyrouth and North Lebanon as else. Emergence of *bla*_OXA-23_ in Lebanon is linked to the clonal spread of the PFGE type A'/ST2. It is noteworthy that we reported in this study the first detection of *bla*_OXA-23_ and *bla*_OXA-24_ in Lebanon. Interestingly, the PFGE type A'/ST2 (harboring *bla*_OXA-66_) dominated heavily in Beyrouth and North Lebanon suggesting an extensively inter-hospital dissemination and thus it could be considered as an epidemic cluster. Beside this epidemic clone, some hospitals had their unique PFGE clone (PFGE type B'/ST2, PFGE type C'/ST2 and PFGE type D'/ST25). In those cases, it is interesting to notice that some clones contained isolates separated by 4 years. These results suggest a successful spread of well-established clones and therefore the urgent need of effective infection control measures to eradicate such bugs.

Our *A. baumannii* strains were analyzed using four epidemiological typing techniques. PFGE was the most discriminating scheme allowing the recognition of 11 types, followed by DiversiLab with 9 types then *bla*_OXA-51_ with 8 types and finally MLST with 7 types.

To our knowledge, there is no sufficient use of adjusted Wallace coefficient in *A. baumannii* typing field, an area which has largely been expanded in the last decades. Many techniques have been proposed with an increasing trend to track this pathogen and assign it to its corresponding international clonal lineage. Therefore, there is an arising need to perform a quantitative comparison between available typing methods using this coefficient or other coefficients to assess their strengths and weaknesses, better understand and validate their fields of application.

PFGE has been considered as a gold standard for outbreak investigations due to its higher discriminatory power which impaired its use for large investigations and population structure studies. As we have shown, PFGE is a very good method to predict *bla*_OXA-51_, MLST and rep-PCR types, but its higher resolution prevents other methods to successfully predict its types. Indeed, PFGE is a time demanding and labor intensive method with many intra and inter laboratory reproducibility problems [27]. *ApaI*, the classical restriction enzyme used for almost *A. baumannii* PFGE protocols generates complex DNA patterns with more than 40 fragments [28]. Chang *et al.*, 2013 [28] suggested the use of two other infrequent cutting restriction enzymes (*Asc*I and *Asi*SI) generating clear patterns with only 10–20 fragments per pattern.

MLST is a highly informative technique which puts isolates in a global context [24] and can directly assign them to their clonal complex. Thus, it is regarded as the method of choice for long term and phylogenetic studies. Although it is a reproducible and portable method, MLST is expensive and time demanding. As expected, MLST was unable to predict PFGE and rep-PCR types because of a discriminatory power lesser than found in PFGE and rep-PCR [12], [24]. Besides, MLST could predict very well (84.7%) all *bla*_OXA-51_ sequences except the colistin resistant strain that belonged to ST2 and had an OXA-336, a single amino acid variant of OXA-66 enzyme.

DiversiLab is a commercial rep-PCR typing system which benefits from several advantages: rapidity with an ability to investigate large number of isolates, standardization, reproducibility with conserved clustering between laboratories [29], and allowing in house libraries building. For *A. baumannii*, rep-PCR had revealed a comparable discriminatory power to PFGE [15]. It has been suggested to study *A. baumannii* population structure with rep-PCR as it can identify eight international clones within 492 isolates from a worldwide collection [7] and generate concordant results with MLST and *bla*_OXA-51_ SBT [16], [30]. Our rep-PCR results showed an overall agreement with PFGE, whenever some exceptions (Fig. 1) have been noticed which explained the low adjust Wallace coefficient (PFGE predicted 69% of rep-PCR types). Compared to PFGE clustering, rep-PCR grouped differently some strains belonging to the same PFGE type or assembled some strains belonging to different PFGE types in the same rep-PCR types, as reported with some other authors [31], [32]. One discrepant result was the strain belonging to ST3 which regrouped with rep-PCR type A, whereas it had a unique PFGE type. When we visually checked the graphs of samples and overlayed functions, we noticed the presence of another peak which was the source of this confusion between strains belonging ST2/rep-PCR type A and strain ST3/rep-PCR type A. Hence, this noticed the importance of careful interpretation of rep-PCR results [31].

The *bla*_OXA-51_ gene is an intrinsic chromosomal beta-lactamase gene naturally found in *A. baumannii* and up to 95 enzyme variants have been identified to date [33]. Sequencing of the entire gene was proposed as a *bla*_OXA-51_ SBT [12]. This SBT could correctly identify the eight international clones characterized by rep-PCR [16]. Also, it correlated well with Bartual's MLST [12]. Compared with Pasteur's MLST, SBT could correctly assign the nine clonal complexes in such manner each CC had specific *bla*_OXA-51_ alleles [17]. We have found similar results, where each CC or ST had a specific *bla*_OXA-51_ variant confirmed by adjusted Wallace coefficient (*bla*_OXA-51_ SBT predict 100% all ST). Indeed, the colistin strain (strain 17) carried OXA-336 which differed only by a single amino acid from OXA-66, indicating that *bla*_OXA-51_ SBT could correctly identify it as belonging to CC2. Compared with PFGE clustering; *bla*_OXA-51_ SBT had a lower discriminatory power. *bla*_OXA-66_ characteristically linked to ST2 has been found in 3 PFGE types (A', B' and C') and one unique profile. The *bla*_OXA-64_ variant carried by ST25 had characteristically been linked to PFGE type D'. Finally, other *bla*_OXA-51-like_ variants had unique PFGE profiles.

## Conclusion

This report describes the first detection of *bla*_OXA-23_ and *bla*_OXA-24_ in Lebanon. Although, our collection is unable to give the real picture of molecular epidemiology in Lebanon, it shed lights on circulating clones and on the mechanisms of carbapenem resistance. Other multi center studies are obviously required to better understand the epidemiology of this bug in the country.

Overall, a good concordance with the four typing methods was shown. PFGE and MLST are reference methods in local and long term epidemiological studies respectively, although both methods are time and cost consuming. *bla*_OXA-51_ SBT seems to be an excellent choice for initial epidemiological screening of isolates. rep-PCR is a rapid tool to access outbreaks at local scale but careful interpretation of results must be done.

## Materials and Methods

### Bacterial strains

A total of 42 non redundant clinical strains of *A. baumannii* isolated from various clinical samples were collected between 2006 and 2012 from the following hospitals: Rafic Harrii Beyrouth governmental hospital, Tripoli governmental hospital (TGH), Nini hospital, Rahal hospital, Monla hospital and Ain Wazein Hospital (AWH). All the bacterial strains were de-identified and a number was attributed prior to access and analysis. No consent was needed since strains used in this study were those isolated during routine analysis in the different laboratories. The clinical sources of the different strains are noted in Table 1.

### Identification

Isolates were routinely cultured on Blood agar at 37°C, and stored at -80°C until further study. Identification to *A. calcoaceticus-baumannii* complex was initially performed using MALDI-TOF Vitek MS (bioMérieux, Marcy-l'Etoile, France) and confirmation of identification at species level was done by real time PCR of *bla*_OXA-51_ gene [34] and partial RNA polymerase b-subunit (*rpoB*) gene sequencing [35].

### Susceptibility testing and investigation of carbapenem resistance mechanisms

Antibiotic Susceptibility testing was determined by the disc diffusion method according to the guidelines of the French Society of Microbiology (www.sfm-microbiologie.org/). Resistance to carbapenem and colistin were confirmed by determining imipenem, meropenem, doripenem and colistin minimum inhibitory concentration (MICs) by Etest strips (bioMérieux, Marcy-l'Étoile, France). Carbapenem resistant isolates were investigated for the presence of carbapenem resistance genes *bla*_OXA-23_, *bla*_OXA-24_, *bla*_OXA-58_, *bla*_OXA-143_, *bla*_ndm-1_ by PCR (Table 3). Presence of the insertion sequence IS*Aba1* was also screened. The association IS*Aba1*-*bla*_OXA-23_ and IS*Aba1*-*bla*_OXA-51_ was tested using a combination of primers IS*Aba1*F with reverse primers targeting *bla*_OXA-23_ or *bla*_OXA-51_ respectively (Table 3).

**Table 3:** Oligonucleotide primers and TaqMan* fluorescent probes used in this study.

Gene	Primer	Primer Sequences	Amplicon size (bp)	References
bla_OXA51-like_	OXA51like-F	5'-AACATTAAAGCACTCTTACTTATAAC	171	[22]
	OXA51like-R	5′-TTGTTGGATAACTAAAACACCCGT		
	OXA51like-Dye	FAM-CTCACCTTATATAGTGTCTGCTAA-BHQ1		
bla_OXA23-like_	OXA23-F1	5′-TGCTCTAAGCCGCGCAAATA	130	[39]
	OXA23-R1	5′-TGACCTTTTCTCGCCCTTCC		
	OXA23-probe	FAM-GCCCTGATCGGATTGGAGAACCA-BHQ1		
bla_OXA24-like_	OXA24-F	5′-CAAATGAGATTTTCAAATGGGATGG	123	[39]
	OXA24-R	5′-TCCGTCTTGCAAGCTCTTGAT		
	OXA24-probe	FAM-GGTGAGGCAATGGCATTGTCAGCA-BHQ1		
bla_OXA58-like_	OXA58-F	5′-CGCAGAGGGGAGAATCGTCT	102	[39]
	OXA58-R	5′-TTGCCCATCTGCCTTTTCAA		
	OXA58-probe	FAM-GGGGAATGGCTGTAGACCCGC- BHQ1		
bla_OXA143-like_	OXA-143-F	5′-TGGCACTTTCAGCAGTTCCT	149	[40]
	OXA-143-R	5′-TAATCTTGAGGGGGCCAACC		
*bla*NDM	NDM-F	5′-GGTGCATGCCCGGTGAAATC	661	[41]
	NDM-R	5′-ATGCTGGCCTTGGGGAACG		
ISAba1	ISAba1	5′-CATTGGCATTAAACTGAGGAGAAA	451	[42]
	ISAba2	5′-TTGGAAATGGGGAAAACGAA		
bla_OXA51-like_	OXA-69A	5′-CTAATAATTGATCTACTCAAG	975	[12]
	OXA-69B	5′-CCAGTGGATGGATGGATAGATTATC		
*rpo*B	Ac696F	5′-TAYCGYAAAGAYTTGAAAGAAG	350	[23]
	Ac1093R	5′-CMACACCYTTGTTMCCRTGA		

*Eurofins MWG Operon, Courtaboeuf, France.

### Epidemiological typing

#### Pulsed field gel electrophoresis (PFGE)

PFGE using *Apa*I as a restriction enzyme was done as described previously [36]. DNA fragments were separated in CHEF-DRIII system (Biorad, Marne LA Coquette, France) at 6V/cm and 14°C for 21 hours with pulse times ranging from 3 s to 20 s. Computer-assisted analysis was performed by using fingerprinting II (Biorad, Marne LA Coquette, France) with the unweighted pair-group method with artithmetic averages (UPGMA) and Dice similarity coefficient for banding pattern comparison. A PFGE type was defined by a cluster of isolates showing ≥80% similarity

#### MLST

MLST was performed according to the Pasteur scheme (http://www.pasteur.fr/recherche/genopole/PF8/mlst/Abaumannii.html). The internal fragments of seven housekeeping genes (*fusA*, *pyrG*, *rpoB*, *rplB*, *cpn60*, *gltA* and *recA*) were amplified, then purified and sequenced by an ABI 3130XL DNA sequencer (Applied Biosystems, Foster City, United States). The sequences were compared to the available sequences present in the MLST website. When a new allele or ST was identified, it was submitted and codified by Institut Pasteur MLST Database. The eBURST [37] was used to compare ST to the existed ST(s) and to assign isolates to their clonal complexes. A clonal complex (CC) is defined as a set of similar ST(s) having 6 identical loci among 7, so a CC is formed by the founder ST and its single locus variants (SLV) [10].

#### rep-PCR

Rep-PCR was performed using the automated system DiversiLab, version 3.4 (bioMérieux, Marcy-l'Étoile, France) following the manufacturer recommendations. Briefly, bacteria were cultured on sheep blood agar (Oxoid). DNA was extracted using the MoBio Ultra Clean microbial DNA extraction Kit, and adjusted to 50 ng/µl. After extraction, DNA was amplified using DiversiLab *Acinetobacter* kit, and the amplified DNA was separated and detected by Agilent 2100 Bioanalyser (Agilent Technologies). The resulted fingerprints were analyzed using the DiversiLab software with the modified Kullback-Leibler (KL) as a statistical method and ≥95% as a threshold to define a cluster of closely related isolates or a rep-PCR type.

#### *bla*_OXA-51_ sequence-based typing (SBT)

This typing method consists to sequence the full length (825 bp) of a single locus *bla*_OXA-51_ gene. The *bla*_OXA-51_ was amplified by external primers OXA-69A/OXA-69B as described [12]. PCR products were purified and sequenced by ABI 3130xl DNA sequencer (Applied Biosystems). The resulted sequences were compared to all variants present in BLAST. When a novel variant was detected, it was submitted to GenBank and assigned by the Lahey database for beta-lactamase classification (http://www.lahey.org/studies/webt.asp).

### Concordance between techniques

The online tool (http://www.comparingpartitions.info/) was used to calculate the adjusted Wallace coefficient. This coefficient is an objective and quantitative measure of clustering agreement between the studied techniques which indicates the probability of a pair of isolates assigned at the same type by one technique is also reassigned at the same by the other technique [32], [38].

### Nucleotide sequence accession numbers and novel sequence types

Two new nucleotide sequences of *bla*_OXA-51_ were submitted to GenBank under accession number KF048907 and KF048908 and assigned respectively by Lahey center as *bla*_OXA-336_ and *bla*_OXA-337_. Two new sequence types were identified and coded by MLST Pasteur as ST284 (3-5-2-1-7-1-4) and ST285 (1-52-2-2-9-4-2). The latter had a new *fusA* allele.

### Ethic statement

Not applicable
